# Investigation of CALML6 expression and its clinical relevance across pan-cancer

**DOI:** 10.1007/s12672-026-05189-5

**Published:** 2026-05-26

**Authors:** Rongchuang Lu, Jingjiu Gu, Yongkui Zhao, Mingyue Guo

**Affiliations:** 1 Department of General Surgery, Chifeng Municipal Hospital, Inner Mongolia Medical University, Chifeng, Inner Mongolia P.R. China; 2 Department of Urinary Surgery, Chifeng Municipal Hospital, Inner Mongolia Medical University, Chifeng, Inner Mongolia P.R. China; 3 Department of Urinary Surgery, Nanjing Drum Tower Hospital, Affiliated hospital of Medical School, Nanjing University, Nanjing, Jiangsu P.R. China; 4 Research Institute of General Surgery, Nanjing Jinling Hospital, Affiliated hospital of Medical School, Nanjing University, Nanjing, Jiangsu P.R. China

**Keywords:** Pan-cancer, Immunological marker, CNV, Methylation, Drug sensitivity

## Abstract

Cancer is a major global health issue, leading to high morbidity and mortality. This study investigates CALML6 expression and its clinical relevance across cancer types using RNA-sequencing data from public databases like UCSC Xena and TCGA, assessing mRNA levels, mutations, CNV, SNV, methylation, and drug sensitivity via GSCA and cBioPortal. Results show CALML6 is upregulated in DLBC, LAML, and THYM, but downregulated in ACC, BRCA, and COAD. Survival analyses link CALML6 expression to DSS, OS, and PFI in ACC, COAD, and KIRC, suggesting its role as a prognostic biomarker. Immune infiltration analysis reveals positive associations with T effector memory and NK CD56bright cells, and negative correlations with immunosuppressive cells. Genetic analysis shows a positive link between CNV and CALML6 mRNA, while methylation and mutation counts are negatively associated. Drug sensitivity analysis indicated that CALML6 expression was correlated with the response patterns of several compounds, although the translational significance of these associations requires further validation. The relative expression levels represented by qRT-PCR validation showed that CALML6 expression was upregulated in BLCA, COAD, and STAD cell lines relative to the corresponding normal cell lines. In conclusion, this study supports the potential relevance of CALML6 as a prognostic biomarker candidate in pan-cancer. Further studies are warranted to validate its biological relevance experimentally and to assess its potential clinical significance in cancer.

## Introduction

Cancer remains one of the leading causes of morbidity and mortality globally, imposing significant healthcare burdens and prompting extensive research into its biological underpinnings and potential therapeutic strategies [1]. With the increasing incidence rates, especially in developing regions, there is an urgent need to identify novel biomarkers that can improve diagnostic and prognostic capabilities in oncology. A promising area of investigation focuses on the role of genes associated with calcium signaling, particularly the calmodulin-like 6 (CALML6) gene, which has shown potential implications in various cancer types due to its involvement in critical cellular processes such as proliferation, apoptosis, and immune response modulation [2]. Current research has established a complex interplay between gene expression and cancer progression, with many studies emphasizing the necessity of understanding specific genetic alterations that contribute to tumorigenesis [3, 4]. Despite advancements in genomic technologies and the availability of large-scale datasets, a knowledge gap persists regarding the precise role of CALML6 in cancer biology. While previous investigations have indicated its involvement in inflammatory responses and cell signaling pathways, comprehensive analyses that elucidate CALML6’s expression patterns across multiple cancer types and its correlation with clinical outcomes remain limited [5].

To bridge this gap, our study employs robust bioinformatics methodologies, utilizing publicly available RNA sequencing data from comprehensive databases such as UCSC Xena (University of California, Santa Cruz Xena Genomic Browser Database) and The Cancer Genome Atlas (TCGA) [6]. By leveraging these extensive datasets, our research aims to characterize the differential expression of CALML6 across various malignancies and assess its prognostic value in relation to disease outcomes. The methodology is designed to ensure a thorough analysis, taking into consideration factors such as sample heterogeneity and clinical parameters, to derive meaningful insights into CALML6’s role as a potential biomarker for cancer prognosis [7].

The primary objective of this study is to explore the clinical significance of CALML6 in the context of multiple cancer types, focusing on its expression levels and the implications for patient prognosis. By systematically analyzing CALML6 expression in conjunction with survival data, our research seeks to provide a comprehensive understanding of its potential as a prognostic biomarker. Furthermore, we aim to explore the biological and clinical associations of CALML6 across cancer types, thereby providing a basis for future mechanistic and translational studies [8].

In summary, this study addresses a critical need within cancer research by investigating the role of CALML6 across diverse malignancies. By employing advanced bioinformatics approaches and large-scale genomic datasets, our work aims to contribute valuable insights into the potential of CALML6 as a biomarker for cancer prognosis, thereby providing a resource for future biological validation and clinical investigation.

## Materials and methods

### Data acquisition and differential analysis

We obtained and analyzed pan-cancer data sets from the UCSC Xena database (https://xenabrowser.net/datapages/) and the RNAseq data in TPM format from the TCGA pan-cancer data set in the GDC database (https://portal.gdc.cancer.gov/). We processed the data using the Toil workflow. We conducted group comparisons and paired sample comparisons of CALML6 expression in pan-cancer.

### Prognostic analysis

We used R packages survival [3.3.1] and ggplot2 [3.4.4] to analyze CALML6’s proportional hazards regression model in diseases using public databases, conducting OS (Overall Survival), DSS (Disease Specific Survival), and PFI (Progression-Free Interval) analyses, comparing the prognostic outcomes of high and low CALML6 groups, and constructing nomograms and calibration curves for pan-cancer. We used the survival package for proportional hazards assumption testing and Cox regression analysis, while the rms package was used to build nomogram-related models and for visualization. The survival package was also used for proportional hazards assumption testing and fitting survival regression, with results visualized using the survminer and ggplot2 packages, creating heatmaps and survival curves for OS, DSS, and PFI. ROC curve analysis was performed using the pROC package in R to evaluate the diagnostic accuracy of CALML6 expression in distinguishing tumor from normal samples in selected cancer types. CALML6 expression was used as the test variable, and sample status (tumor vs. normal) was used as the binary outcome. The area under the curve (AUC) was calculated to assess diagnostic discrimination. Proportional hazards assumption testing and fitting survival regression were conducted to analyze and compare prognostic outcomes between high and low CALML6 groups across various pan-cancer subgroups.

### Enrichment analysis

We searched for CALML6 on the STRING website (https://cn.string-db.org/) to obtain related hub genes. These related genes were analyzed for Gene Ontology (GO) and Kyoto Encyclopedia of Genes and Genomes (KEGG) with the GOKEGG package, followed by enrichment analysis and Gene Set Enrichment Analysis (GSEA) using the GSEA package, and the correlation between pairs of variables in the data was visualized using the circlize package.

### Immune infiltration analysis

We analyzed the correlation between single genes and immune infiltration results, using estimate, Cibersort, and ssGSEA to assess the immune infiltration status of the data. We analyzed the correlation between the main variables and the immune infiltration matrix, and examined the differences in immune infiltration results between high and low expression groups based on public data.

### Gene alterations

We examined mRNA expression of CALML6, mutations, copy number variation (CNV), single nucleotide variation (SNV), methylation, and drug sensitivity on the GSCA website (https://guolab.wchscu.cn/GSCA). Additionally, we analyzed CALML6 gene mutations on the cBioPortal website (http://www.cbioportal.org/results/plots). We also looked at how CALML6 gene copy number relates to mRNA, mutation counts, and putative copy-number alterations from GISTIC, as well as Admixture% EUR: Genetic Ancestry, phosphoprotein site level expression data by CPTAC, and methylation [9, 10].

### Quantitative real-time PCR(qRT-PCR)

Total RNA was extracted from commercially obtained BLCA, COAD, and STAD cancer cell lines and their corresponding normal cell lines. Quantitative PCR was performed using SYBR Green chemistry on an Applied Biosystems 7500 Fast Real-Time PCR System (Applied Biosystems, USA). The primer sequences were as follows: CALML6 forward 5′-GGGCTACATTGACTGGAACACAC-3′ and reverse 5′-CCTCATAGTCGATGGTCCTGTC-3′ ; GAPDH forward 5′-GTCTCCTCTGACTTCAACAGCG-3′ and reverse 5′-ACCACCCTGTTGCTGTAGCCAA-3′. GAPDH was used as the internal reference gene. The cycling conditions were: 95 °C for 10 min, followed by 40 cycles of 95 °C for 15 s and 60 °C for 60 s, and a melting curve analysis was performed at the end of amplification to confirm product specificity. Relative expression levels were calculated using the 2^-ΔΔCt method.

### Analysis of *CALML6* dependency in cancer cell lines

To evaluate the potential functional essentiality of CALML6 across different tumor types, we utilized the large-scale CRISPR-Cas9 screening data from the Cancer Dependency Map (DepMap) portal (https://depmap.org/portal/, Public 25Q3 release). The CRISPR gene effect scores were calculated using the Chronos algorithm, where a score of 0 indicates no effect on cell viability, and a score of -1.0 resembles the effect of knocking out a pan-essential gene. A Chronos score < -0.5 was considered to indicate gene dependency. Additionally, *CALML6* mRNA expression data (Log2 TPM + 1) for the corresponding cell lines were extracted to investigate the relationship between gene expression levels and functional dependency. The Pearson correlation coefficient was calculated to assess the linear correlation between *CALML6* expression and its gene effect scores. All visualizations and statistical analyses were performed using R software with the “ggplot2” and “ggpubr” packages.

### Data integration, preprocessing, and quality control

Data for the present study were obtained from multiple public platforms, and each database was used for a specific analytical purpose rather than for direct pooled modeling of raw cross-platform data. Expression data, survival data, and clinicopathological information were mainly obtained from TCGA/UCSC Xena. UCSC Xena-provided TPM expression matrices were used for expression-related analyses. GSCA was used for analyses related to CNV, SNV, methylation, and drug sensitivity. cBioPortal was used to obtain an overview of genomic alterations and for complementary validation of alteration-related findings. DepMap/CRISPR data were used for dependency analysis. Immune infiltration analyses were performed using ssGSEA-based approaches according to the corresponding processed expression data.

For all analyses, processed or normalized data provided by the corresponding database platforms were used. GSCA and cBioPortal analyses were based on the standardized data available within those platforms. Raw expression values from different databases were not directly merged for joint cross-platform modeling. Instead, each analytical module was conducted independently according to data availability within the corresponding database, and the results from different platforms were used in a complementary manner for cross-validation and biological interpretation.

For quality control, only samples with available expression data and relevant clinical or survival information were included in the corresponding analyses. Samples lacking key variables required for a given module were excluded from that specific analysis. The conclusions of this study were therefore drawn from convergent observations across complementary analytical modules rather than from direct cross-platform pooling of raw data.

### Statistical analysis

All statistical analyses were performed using R software (version 4.2.1). For normally distributed data, Student’s t-test was used; for non-normally distributed data, the Wilcoxon rank-sum test was applied; and for comparisons among two or more groups, the Kruskal–Wallis test was used. Spearman correlation analysis was used to evaluate associations between variables. To account for multiple testing and reduce the risk of false-positive findings, p-values were adjusted to control the false discovery rate (FDR). FDR correction was performed separately within each analytical module, including differential expression analyses, survival analyses, clinicopathological subgroup comparisons, immune infiltration analyses, genomic and epigenetic analyses, drug sensitivity analyses, and dependency- and pathway-related correlation analyses. An adjusted p-value (FDR) < 0.05 was considered statistically significant.

## Results

### Differences in CALML6 expression across pan-cancer

**Fig. 1:**
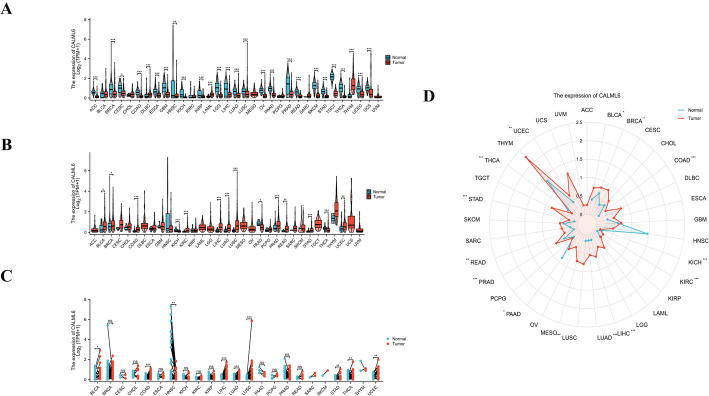
Differential Expression of CALML6. **A** Violin plot showing CALML6 mRNA expression in 33 tumors from TCGA-GTEx samples. **B** Violin plot showing CALML6 mRNA expression in 33 tumors from TCGA samples. **C** Violin plot showing CALML6 mRNA expression in 18 paired tumor samples from TCGA data. **D** Radar chart of CALML6 mRNA expression in 33 tumors from TCGA samples

Data analysis using the UCSC Xena database indicates that CALML6 is significantly upregulated in several tumors, such as DLBC, LAML, and THYM. On the other hand, it is significantly downregulated in many tumors: ACC, BRCA, COAD, ESCA, GBM, HNSC, KICH, KIRC, KIRP, LGG, LIHC, LUAD, LUSC, OV, PAAD, PRAD, READ, SKCM, STAD, TGCT, THCA, UCEC, and UCS (Fig. 1A). Subsequently, analysis using the TCGA database showed that CALML6 expression differs in various cancers compared to adjacent normal tissues, including BLCA, BRCA, COAD, HNSC, KIRC, LIHC, LUAD, LUSC, PRAD, READ, STAD, and UCEC (Fig. 1B). In paired samples, we observed significant expression differences in BLCA, COAD, HNSC, LIHC, LUAD, LUSC, STAD, THCA, and UCEC (Fig. 1C). Next, we created a radar chart showing CALML6 expression across pan-cancer data from the TCGA database, indicating significant differences in tumors such as BLCA, BRCA, KICH, KIRC, LIHC, LUAD, MESO, PAAD, PRAD, READ, STAD, THCA, and UCEC (Fig. 1D). These results indicate that CALML6 expression differs substantially across cancer types, suggesting potential context-dependent biological and clinical relevance.

### Prognostic significance of CALML6 gene expression across various cancer types

In terms of prognostic impact, we found that high expression of CALML6 was associated with shortened PFS, DSS and OS in patients with ACC, COAD and LGG (all *p* < 0.05). However, for GBM and KIRC, only DSS and OS were shortened, showing statistical differences. That is to say, for these tumors, CALML6 is a risk factor. Conversely, this gene was negatively correlated with DSS and OS shortening in LUSC, and negatively correlated with OS in STAD, indicating that it is a protective factor for LUSC and STAD.

**Fig. 2:**
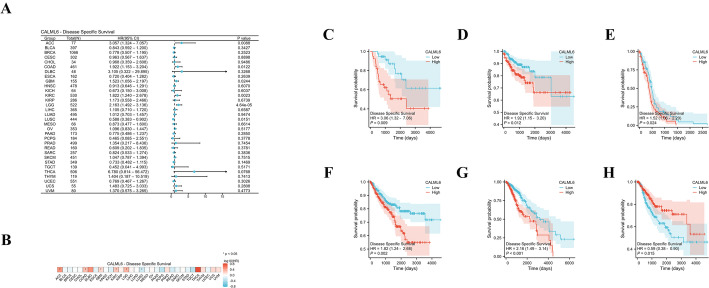
DSS Prognostic Analysis of CALML6 in Pan-Cancer. **A** Forest plot of DSS prognostic analysis created based on COX regression analysis, presented as HR (95% confidence interval). CALML6 is a risk factor in tumors with HR greater than 1 and a protective factor in tumors with HR less than 1. **B** Heatmap of DSS prognostic analysis created based on COX regression analysis. Red indicates risk factors, blue indicates protective factors. * indicates *p* < 0.05, ** indicates *p* < 0.01, *** indicates *p* < 0.001. **C**–**H** KM survival curves of CALML6 in pan-cancer created based on COX regression analysis. The above shows KM survival curves with statistically significant differences

We plotted heatmaps and forest plots for DSS of patients with 33 types of tumors, indicating significant differences in CALML6 expression among various tumors. The 95% confidence interval for ACC is (1.324–7.057), with a Hazard Ratio of 3.057. For COAD, the 95% confidence interval is (1.153–3.204), with a Hazard Ratio of 1.992; for KIRC, the 95% confidence interval is (1.240–2.678), with a Hazard Ratio of 1.822; and for LGG, the 95% confidence interval is (1.429–3.136), with a Hazard Ratio of 2.163. We also plotted Kaplan-Meier (KM) curves for CALML6 in these tumors. In ACC, COAD, GBM, KIRC, LGG, and LUSC, CALML6 significantly affects patients’ DSS (Fig. 2).

**Fig. 3:**
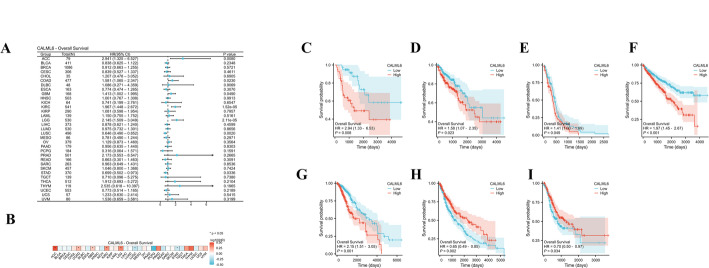
OS Prognostic Analysis of CALML6 in Pan-Cancer. **A** Forest plot of OS prognostic analysis created based on COX regression analysis, presented as HR (95% confidence interval). CALML6 is a risk factor in tumors with HR greater than 1 and a protective factor in tumors with HR less than 1. **B** Heatmap of OS prognostic analysis created based on COX regression analysis. Red indicates risk factors, blue indicates protective factors. * indicates *p* < 0.05, ** indicates *p* < 0.01, *** indicates *p* < 0.001. **C**–**I** KM survival curves of CALML6 in pan-cancer created based on COX regression analysis. The above shows KM survival curves with statistically significant differences

We plotted heatmaps and forest plots for OS of patients in these tumors, with significant differences observed in ACC, COAD, GBM, KIRC, LGG, LUSC, and STAD. We also plotted KM curves for CALML6 in these tumors. In ACC, COAD, GBM, KIRC, LGG, LUSC, and STAD, CALML6 significantly affects patients’ OS (Fig. 3).

**Fig. 4:**
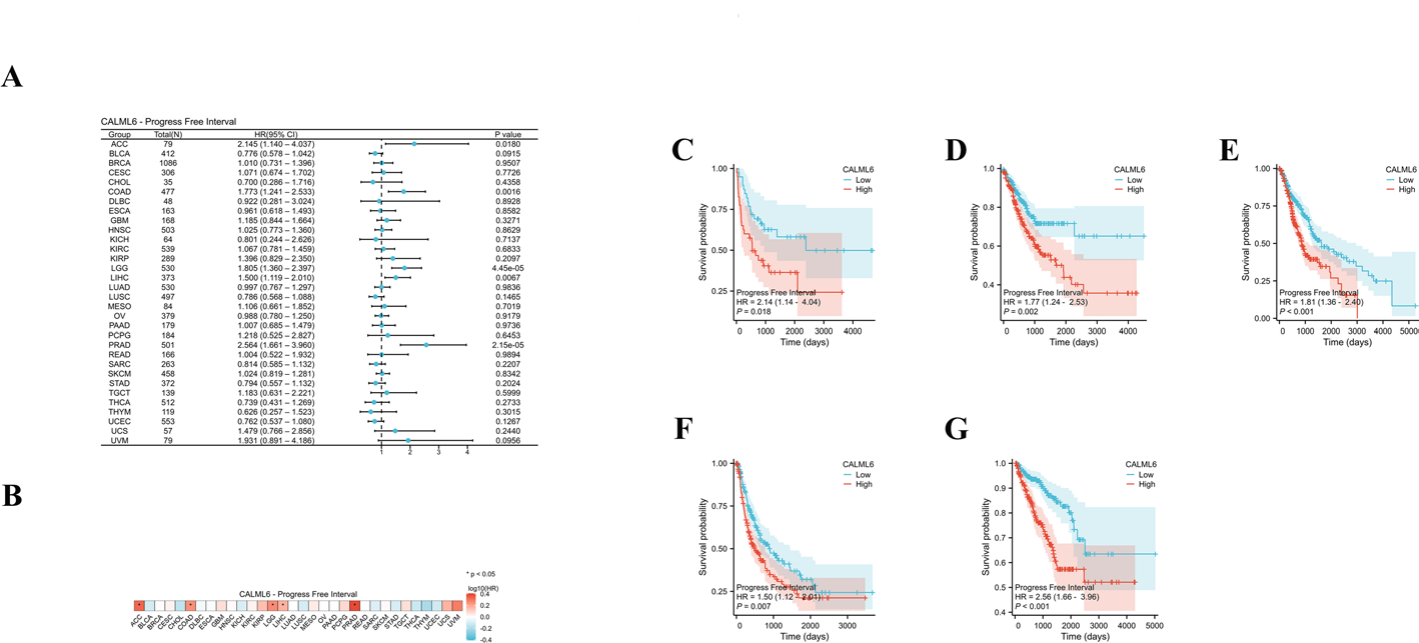
PFI Prognostic Analysis of CALML6 in Pan-Cancer. **A** Forest plot of PFI prognostic analysis created based on COX regression analysis, presented as HR (95% confidence interval). CALML6 is a risk factor in tumors with HR greater than 1 and a protective factor in tumors with HR less than 1. **B** Heatmap of PFI prognostic analysis created based on COX regression analysis. Red indicates risk factors, blue indicates protective factors. * indicates *p* < 0.05, ** indicates *p* < 0.01, *** indicates *p* < 0.001. **C**–**G** KM survival curves of CALML6 in pan-cancer created based on COX regression analysis. The above shows KM survival curves with statistically significant differences

We plotted heatmaps and forest plots for PFI of patients in pan-cancer, with significant differences in ACC, COAD, LGG, LIHC, and PRAD. We also plotted KM curves for CALML6 in these tumors. In ACC, COAD, LGG, LIHC, and PRAD, CALML6 significantly affects patients’ PFI (Fig. 4). The KM analysis determined that higher CALML6 expression reduced OS, DSS, and PFI in ACC, COAD, and LGG, and also shortened OS and DSS in KIRC and GBM, as well as PFI in LIHC and PRAD. In LUSC, it improved DSS and OS, and improved OS in STAD (Figs. 2, 3 and 4).

**Fig. 5:**
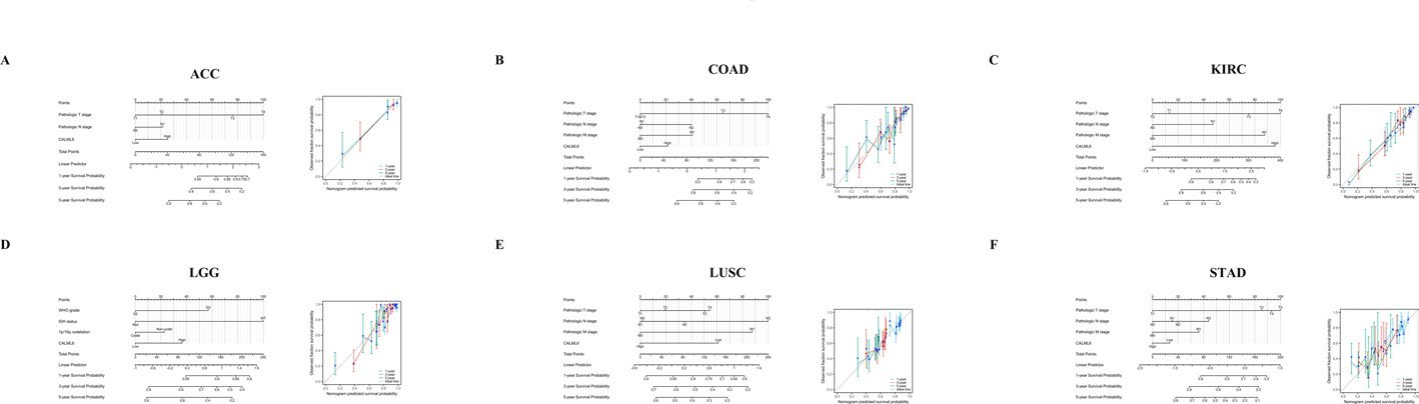
Construction of Clinical Prognostic Model and Calibration Curves Based on High and Low Groups of CALML6. **A** In ACC, pathological T grade, pathological N grade, and high expression of CALML6 can lower patients’ 1-, 3-, and 5-year survival rates. The nomogram and corresponding calibration curve constructed based on this. **B** In COAD, pathological T grade, pathological N grade, pathological M grade, and high expression of CALML6 can lower patients’ 1-year, 3-year, and 5-year survival rates. The nomogram and corresponding calibration curve constructed based on this. **C** In KIRC, pathological T grade, pathological N grade, pathological M grade, and high expression of CALML6 can lower patients’ 1-, 3-, and 5-year survival rates. The nomogram and corresponding calibration curve constructed based on this. **D** In LGG, WHO grade, IDH status, 1p/19q codeletion, and high expression of CALML6 can lower patients’ 1-, 3-, and 5-year survival rates. The nomogram and corresponding calibration curve constructed based on this. **E** In LUSC, pathological T grade, pathological N grade, pathological M grade, and high expression of CALML6 can improve patients’ 1-, 3-, and 5-year survival rates. The nomogram and corresponding calibration curve constructed based on this. **F** In STAD, pathological T grade, pathological N grade, pathological M grade, and high expression of CALML6 can improve patients’ 1-, 3-, and 5-year survival rates. The nomogram and corresponding calibration curve constructed based on this

**Fig. 6:**
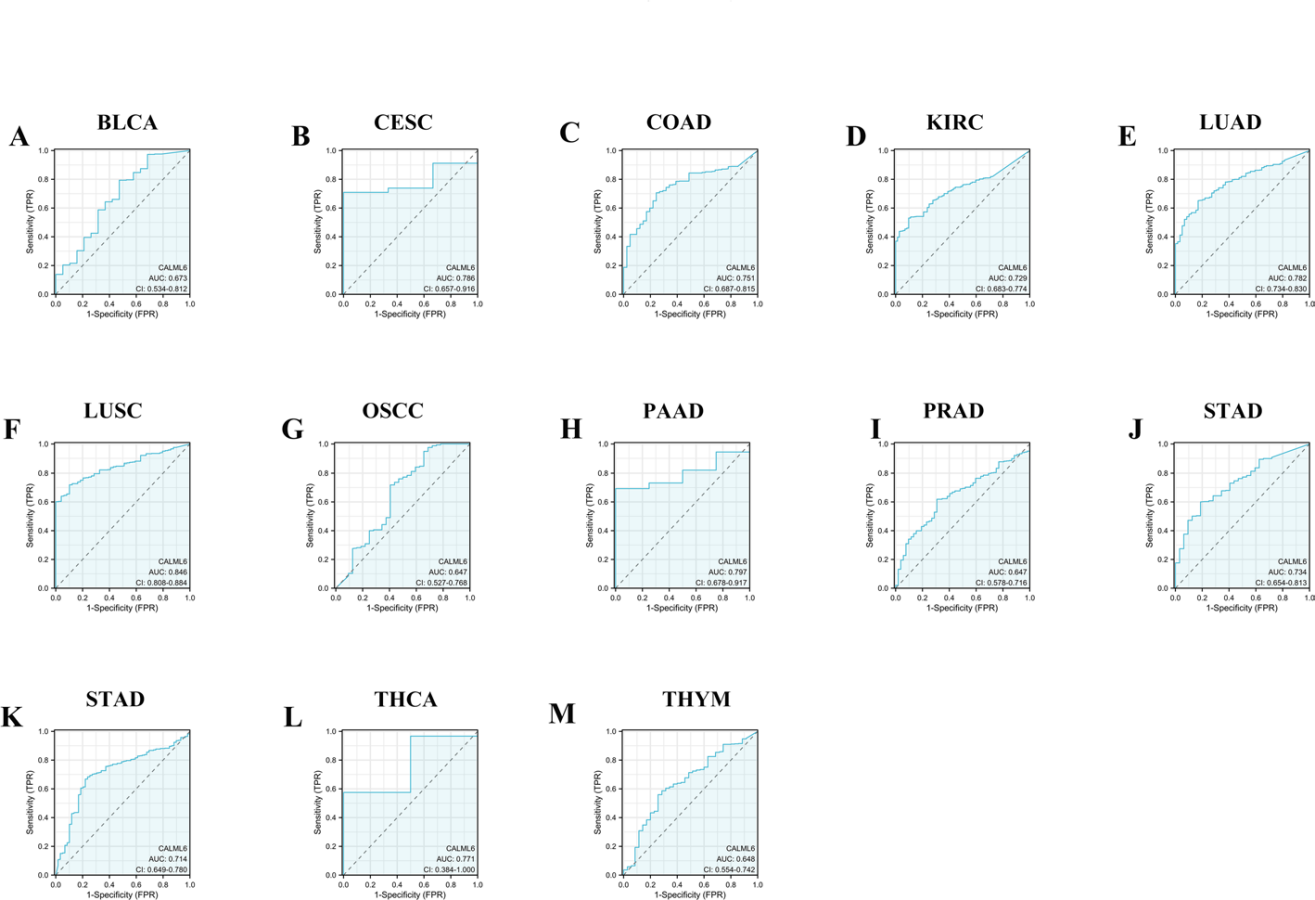
ROC curves of CALML6 expression for distinguishing tumor from normal samples in selected cancer types. Only cancer types with an AUC > 0.6 are shown. **A**–**M** ROC curves for BLCA, CESC, COAD, KIRC, LUAD, LUSC, OSCC, PAAD, PRAD, STAD, THCA, and THYM

We further evaluated the prognostic significance of CALML6 in TCGA pan-cancer cohorts by constructing Cox regression models and nomograms. Patients were stratified into high- and low-expression groups to predict 1-, 3-, and 5-year DSS, OS, and PFI in ACC, COAD, KIRC, LGG, LUSC, and STAD, and the corresponding calibration curves (Fig. 5). Nomograms and corresponding calibration curves were constructed for ACC, COAD, KIRC, LGG, LUSC, and STAD based on CALML6 expression and clinicopathological variables (Fig. 5). ROC curve analysis was further performed to assess the ability of CALML6 expression to distinguish tumor from normal samples in selected cancer types. Only cancer types with an AUC > 0.6 are presented in Fig. 6. In BLCA, CESC, COAD, KIRC, LUAD, LUSC, OSCC, PAAD, PRAD, STAD, THCA, and THYM, the AUC values ranged from 0.647 to 0.864, indicating moderate to good discriminatory performance. In BLCA (AUC = 0.673), CESC (AUC = 0.786), COAD (AUC = 0.751), KIRC (AUC = 0.729), LUAD (AUC = 0.782), LUSC (AUC = 0.864), OSCC (AUC = 0.647), PAAD (AUC = 0.797), PRAD (AUC = 0.647), STAD (AUC = 0.714), THCA (AUC = 0.771), THYM (AUC = 0.648). A larger AUC value indicates better discriminatory performance. Tumors with an AUC greater than 0.6 are displayed here (Fig. 6).

### Clinical associations of CALML6 across diverse cancer types

**Fig. 7:**
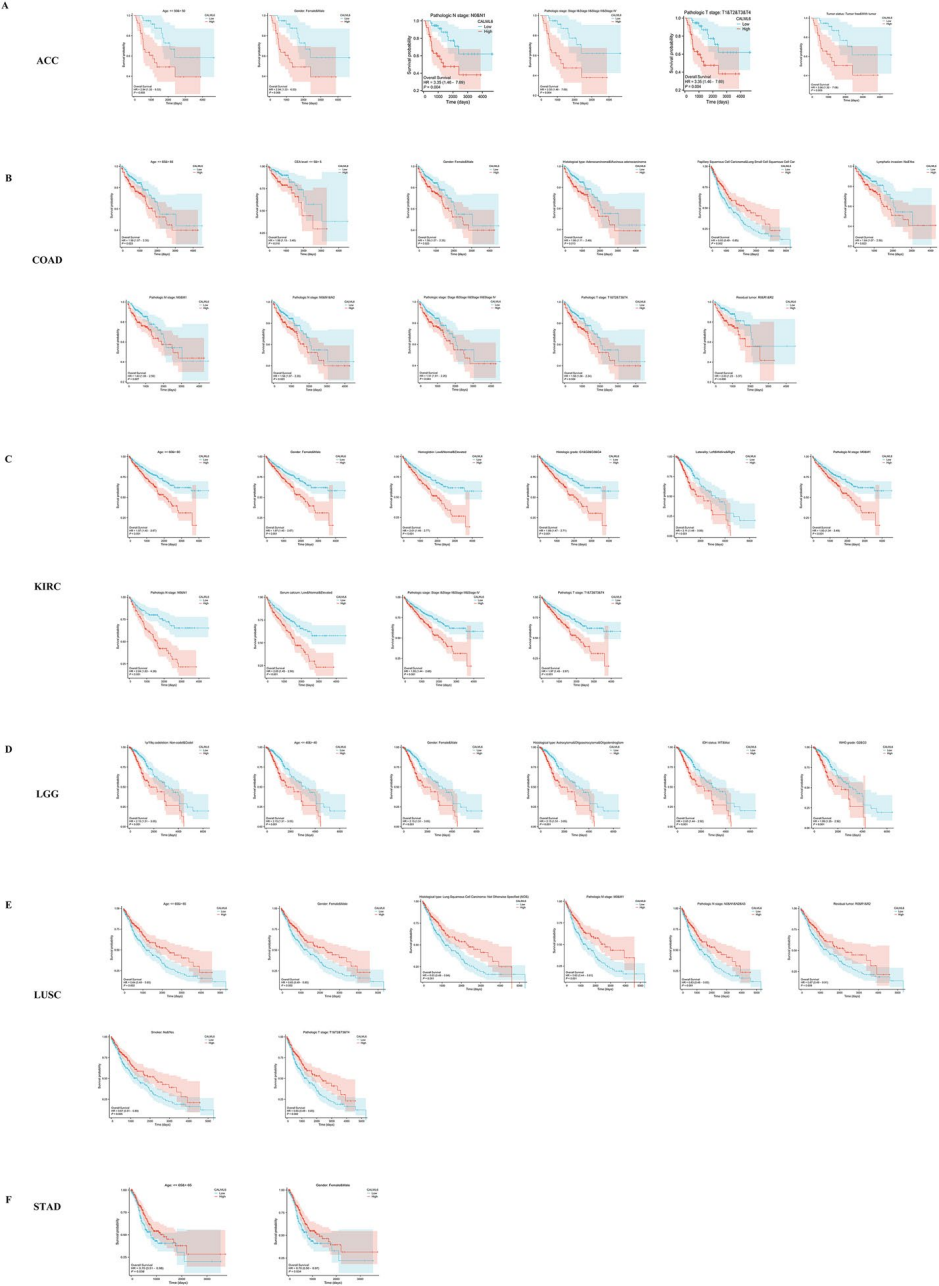
KM Survival Curves of Subgroups of CALML6 in Pan-Cancer. **A** In the ACC subgroup, CALML6 shows significant differences in expression based on patient age, sex, pathological T stage, N stage, pathological grade, and tumor status. **B** In the COAD subgroup, CALML6 shows significant differences in expression based on patient age, sex, CEA level, pathological T stage, N stage, M stage, pathological stage, pathological type, pathological subtype, lymphatic invasion, and residual tumor. **C** In the KIRC subgroup, CALML6 shows significant differences in expression based on patient age, sex, hemoglobin level, pathological T stage, N stage, M stage, pathological stage, histological grade, and laterality. **D** In the LGG subgroup, CALML6 shows significant differences in expression based on patient age, sex, 1p/19q codeletion, pathological type, IDH status, and WHO grade. **E** In the LUSC subgroup, CALML6 shows significant differences in expression based on patient age, sex, CEA level, pathological T stage, N stage, M stage, pathological type, residual tumor, and smoker status. **F** In the STAD subgroup, CALML6 shows significant differences in expression based on patient age and sex

We compared CALML6 expression across tumor subgroups in pan-cancer. Among these, CALML6 expression in the pan-cancer subgroup shows significant differences in age and gender. Additionally, in the ACC subgroup, significant differences were found in pathological N, stage, and T stages. CALML6 shows significant differences in pathological T stage, pathological N stage, pathological M stage, CEA level, pathological type, pathological subtype, lymphatic invasion, and residual tumor. In the KIRC subgroup, significant differences were found in histological grade, laterality, pathological N stage, pathological M stage, serum calcium, and pathological stage. In the LGG subgroup, significant differences were noted in 1p/19q codeletion, pathological type, Isocitrate Dehydrogenase (IDH) status, and World Health Organization (WHO) grade. In the LUSC subgroup, significant differences were found in pathological type, residual tumor, pathological T stage, pathological N stage, pathological M stage, and smoking status. In STAD, no significant differences were found in expressions other than age and gender in other subgroups (Fig. 7). These results suggest that the expression of CALML6 has an impact on the prognosis of many patients and can be considered as a prognostic indicator for these tumors.

### Enrichment analysis of hub genes

**Fig. 8:**
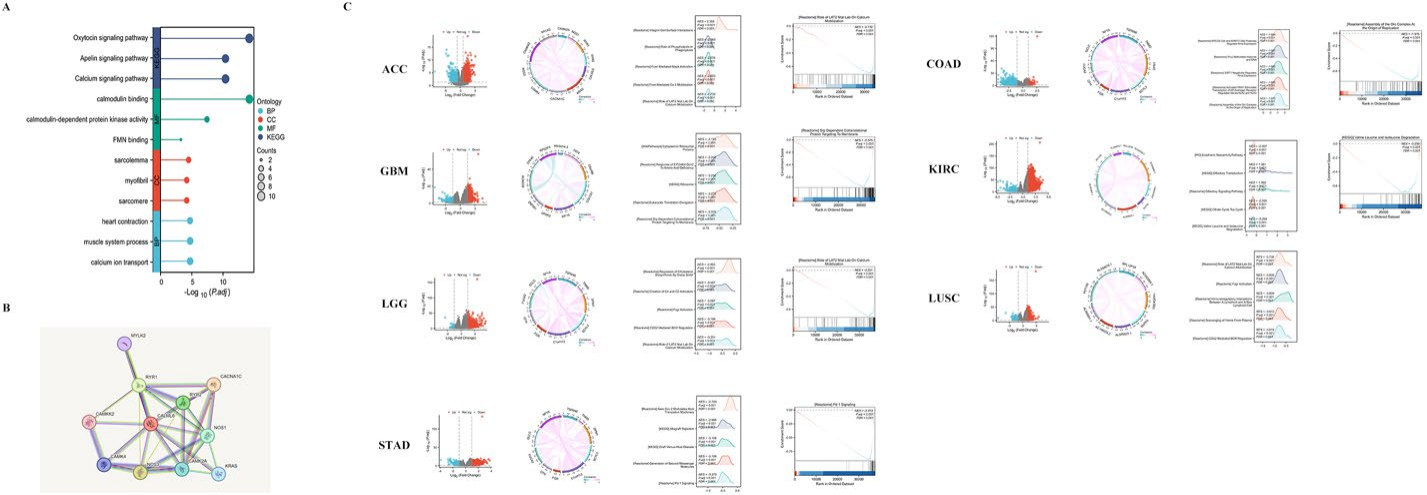
Functional Enrichment Analysis of CALML6-Related Hub Genes. A: Bubble chart of GOKEGG enrichment. Divided into four modules: BP, CC, MF, and KEGG; the larger the bubble, the greater the number of enrichments. B: PPI chart of CALML6-related hub genes, showing the top 10 genes correlated with CALML6. C: Volcano plot of differential expression of CALML6 in ACC, GBM, COAD, KIRC, LGG, LUSC, STAD, along with chord diagrams, GSEA enrichment mountain plots, and advanced visualization charts

On the STRING website, we found genes that are significantly correlated with CALML6. We conducted GOKEGG analysis on these genes and visualized the findings, presenting only the top three pathways with significant differences. The three pathways with the most enrichment and significant differences are: Oxytocin signaling pathway, Apelin signaling pathway, and Calcium signaling pathway (Fig. 8A). We downloaded the PPI interaction diagram from the STRING website (Fig. 8B). We then analyzed single-gene expression difference data and plotted a volcano plot in pan-cancer, along with a chord diagram of correlated genes, and conducted GSEA enrichment analysis, producing a mountain plot and advanced visualization (Fig. 8C). Further studies on these signaling pathways and the enriched cellular phenotypes will help us to further explore the mechanism of CALML6 in the occurrence and development of various cancers.

### Immune infiltration analysis of CALML6

**Fig. 9:**
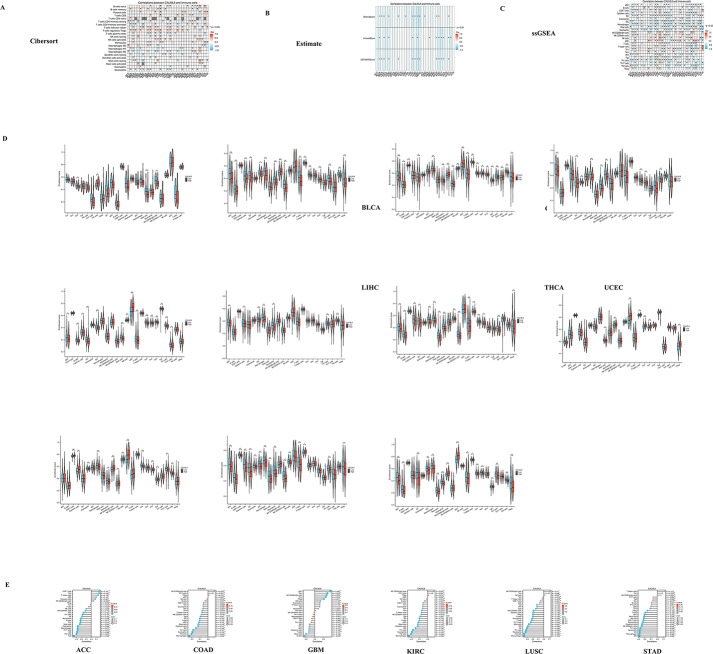
Correlation Analysis of CALML6 and Immune Infiltration. **A** Results of correlation analysis between CALML6 and immune infiltration based on the Cibersort algorithm using public gene data, displayed in heatmap form. The stronger the positive correlation, the redder the squares; the stronger the negative correlation, the bluer they get. * indicates < 0.05. **B** Results of correlation analysis between CALML6 and immune infiltration based on the Estimate algorithm using public gene data, displayed in heatmap form. The stronger the positive correlation, the redder the squares; the stronger the negative correlation, the bluer they get. * indicates < 0.05. **C** Results of correlation analysis between CALML6 and immune infiltration based on the ssGSEA algorithm using public gene data, displayed in heatmap form. The stronger the positive correlation, the redder the squares; the stronger the negative correlation, the bluer they get. * indicates < 0.05. **D** Differences in immune infiltration results between high and low expression groups of CALML6 (median) across 24 types of cancer based on public data. **E** Analysis of the correlation results between CALML6 and immune infiltration, presented in lollipop chart form

In order to investigate the role of CALML6 in immune regulation, we examined the correlation between CALML6 and immune infiltration in all tumors in TCGA. Almost all tumors showed a varying degree of correlation with immune infiltration. CALML6 is linked to immune infiltration and immunotherapy across various cancers. Using the TIMER2 database, we analyzed the correlation between CALML6 expression and immune cell infiltration in pan-cancer using three algorithms: Cibersort, estimate, and ssGSEA, and plotted heatmaps of CALML6 immune infiltration in pan-cancer (Fig. 9A, B, C). We conducted a correlation analysis between the main variables and the immune infiltration matrix data in these tumor datasets. Based on the public data analysis of molecular expression (median) levels, we identified the differences in the immune infiltration results of 24 types of immune cells between the high and low expression groups. We then selected some cancers with significant differences for presentation (Fig. 9D). We then plotted bar charts of immune infiltration correlations for ACC, COAD, GBM, KIRC, LUSC, and STAD. The results show that CALML6 expression is positively correlated with Tem and NK CD56bright cells in various cancers, while negatively correlated with iDC, Th1 cells, and Neutrophils (Fig. 9E). These findings suggest that CALML6 may be associated with tumor immune contexture and may warrant further evaluation as a candidate biomarker in immunotherapy-related studies.

### Gene alterations of CALML6: CNV, SNV, and methylation patterns in pan-cancer analysis

**Fig. 10:**
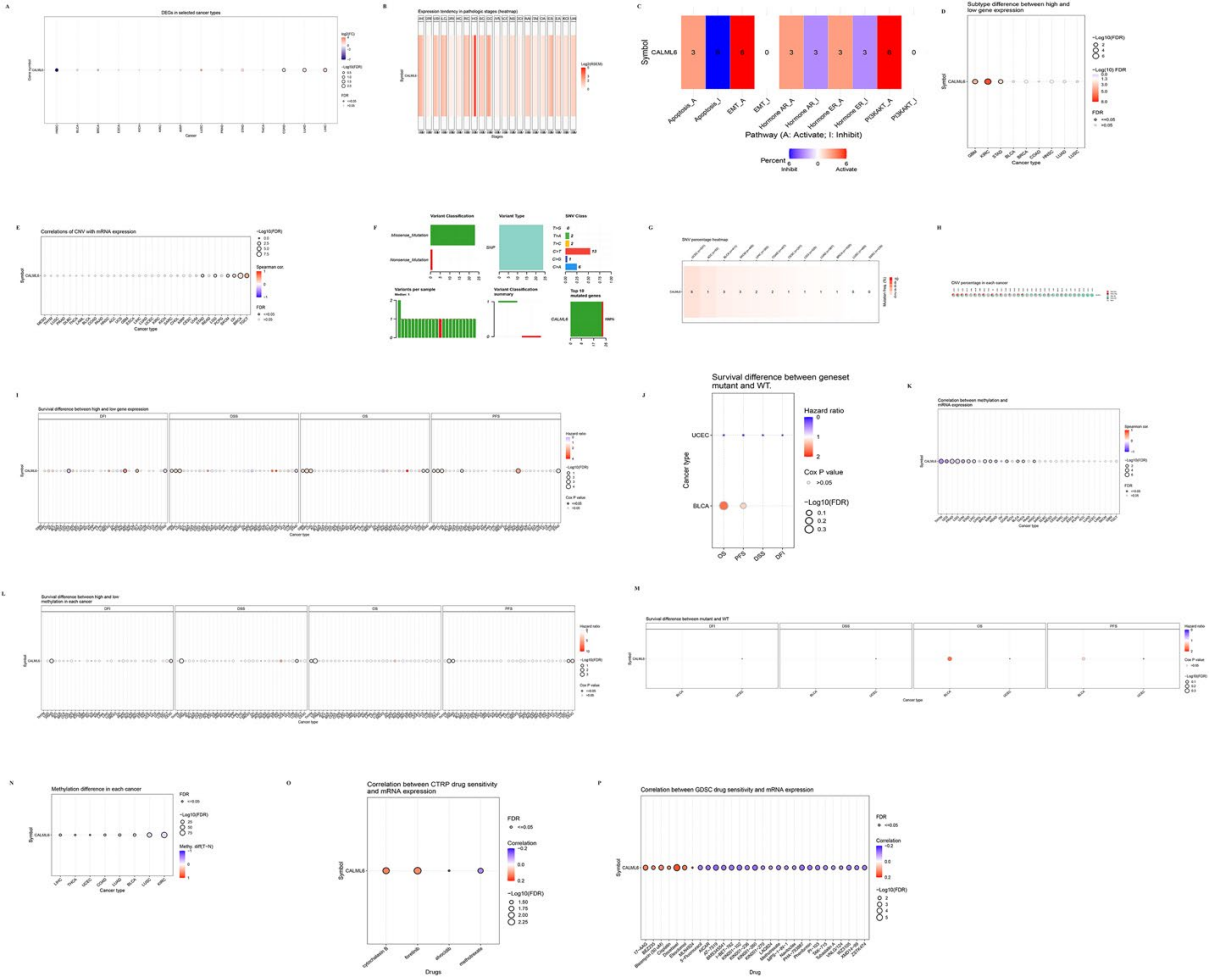
Analysis of CALML6-Related Genes and Gene Mutations on GSCA Website. **A** Bubble chart of differential gene expression of CALML6 in pan-cancer. Displays tumors with significant differences (*p* < 0.05). **B** Heatmap of CALML6 expression in pan-cancer pathological stages. **C** Pathways and phenotypes enriched by CALML6. **D** Expression differences of CALML6 in different tumor subtypes. **E** Correlation between CNV and CALML6 mRNA expression. **F** Variant classification, variant type, SNV class, variants per sample, variant classification summary, top 10 mutated genes. **G** Heatmap of SNV percentage. **H** CNV percentage in each cancer. **I** Survival difference between high and low gene expression (EFI, DSS, OS, PFS). **J** Survival difference between gene set mutant and WT (EFI, DSS, OS, PFS) shown in bubble chart. **K** Correlation between methylation and mRNA expression in different cancers. **L** Survival difference between high and low methylation in each cancer (EFI, DSS, OS, PFS). **M** Survival difference between mutant and WT (EFI, DSS, OS, PFS). **N** Methylation difference in each cancer. **O** Correlation between CTRP drug sensitivity and mRNA expression. **P** Correlation between GDSC drug sensitivity and mRNA expression

In order to investigate the influence of gene mutations on gene expression, we analyzed the mutation status of the CALML6 gene in different tumors. We reviewed the genetic variations of the CALML6 gene in various cancers. It was found that in many cases, these genes have varying degrees of correlation with copy number variations (CNV), single nucleotide variations (SNV), and methylation. We examined Differential Expression Genes (DEGs) in pan-cancer using GSCA (Fig. 10A). We then reviewed the heatmap of CALML6 expression across pathological stages in pan-cancer (Fig. 10B), the signal pathway diagram of gene enrichment (Fig. 10C), and CALML6 expression in subtypes (Fig. 10D). We found a bubble chart showing the correlation between CALML6 CNV and mRNA expression in pan-cancer (Fig. 10E). Next, we reviewed the classification of SNV mutations, types, median number of variations in samples, summary charts of mutation classifications, and the top 10 mutated genes (Fig. 10F). We downloaded the percentage heatmap of CNV mutations in pan-cancer (Fig. 10G) and a dot plot (Fig. 10H). We also obtained a bubble chart illustrating survival differences in gene expression (Fig. 10I), with many tumors showing significant differences, such as GBM, KIRC, LGG, ACC, BLCA, CESC, CHOL, KIRP, MESO, PRAD, TGCT, THCA, LUSC, STAD, etc. Subsequently, we reviewed the bubble chart of survival differences between mutant and WT genes (Fig. 10J), with the highest frequency appearing in BLCA and UCEC. The correlation between CALML6 gene methylation and mRNA expression is positively correlated in tumors such as THYM, UCS, PRAD, LGG, UVM, LIHC, BRCA, KIRP, READ, COAD, BLCA, THCA, and HNSC (Fig. 10K). However, only PRAD, PAAD, TGCT, CESC, LGG, THCA, LUSC, and THYM have significant impacts on prognosis (Fig. 10L). The prognosis of mutant and WT only affects BLCA and UCEC (Fig. 10M). Finally, we reviewed the differences in methylation in pan-cancer, with significant differences found in LIHC, THCA, UCEC, COAD, LUAD, BLCA, LUSC, and KIRC (Fig. 10N). Here, we analyzed the correlation between CALML6 mRNA expression and drug sensitivity using the Cancer Therapeutics Response Portal (CTRP) and Genomics of Drug Sensitivity in Cancer (GDSC) tumor drug sensitivity databases, producing dot plots (Fig. 10O and P). In the CTRP drug sensitivity database, drugs significantly correlated with CALML6 gene expression include cytochalasin B, foretinib, albocidib, and methotrexate. In GDSC, many drugs significantly correlated with CALML6 gene expression were found, with positive correlations including Docetaxel, 17-AAG, Bleomycin, and BEZ235, while negative correlations include AT-7519, BMS345541, methotrexate, and PHA-793,887.

**Fig. 11:**
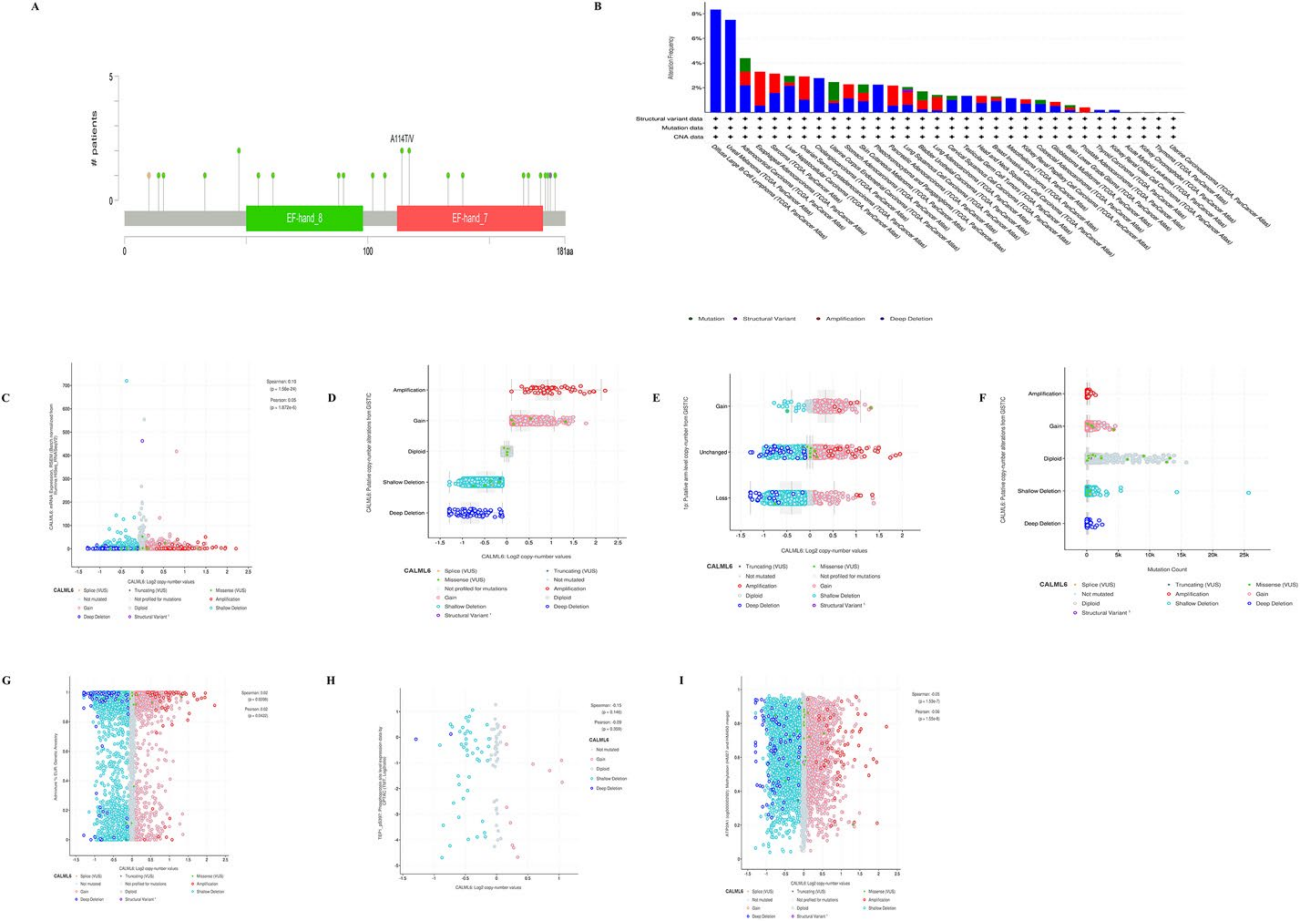
Analysis of CALML6-Related Genes and Gene Mutations in cBioPortal Database. **A** Specific mutation site map of sequencing samples. Circles in the mutation map are colored by mutation type. Mutation types and corresponding color codes are as follows: - Missense Mutations (putative driver) - Missense Mutations (unknown significance) - Truncating Mutations (putative driver): Nonsense, Nonstop, Frameshift deletion, Frameshift insertion, Splice site - Truncating Mutations (unknown significance): Nonsense, Nonstop, Frameshift deletion, Frameshift insertion, Splice site - Inframe Mutations (putative driver): Inframe deletion, Inframe insertion - Inframe Mutations (unknown significance): Inframe deletion, Inframe insertion - Splice Mutations (putative driver) - Splice Mutations (unknown significance) - Fusion Mutations - Other Mutations (putative driver): All other types of mutations - Other Mutations (unknown significance): All other types of mutations. **B** Statistical results of the proportion of different omics alteration types of each gene in pan-cancer samples. **C**–**I** Correlation between CALML6 gene copy number and mRNA, mutation counts, CALML6: putative copy-number alterations from GISTIC, 1p: putative copy-number alterations from GISTIC Admixture% EUR: Genetic Ancestry, phosphoprotein site level expression data by CPTAC, and methylation. The figure notes the correlation and p-values calculated by both Spearman and Pearson methods. Different colored circles represent different genetic alterations

On the cBioPortal website, we first observed the mutation locations of sequencing samples. The main mutation patterns of CALML6 are Missense and fusion (Fig. 11A). We analyzed the copy number of the CALML6 gene and its mRNA expression (Spearman: 0.10, *p* = 1.56e-24, Pearson: 0.05, *p* = 1.872e-6), Admixture% EUR: Genetic Ancestry (Spearman: 0.02, *p* = 0.0208, Pearson: 0.02, *p* = 0.0422), phosphoprotein site level expression data by CPTAC (Spearman: -0.15, *p* = 0.146, Pearson: -0.09, *p* = 0.359), Methylation (Spearman: -0.05, *p* = 1.53e-7, Pearson: -0.06, *p* = 1.55e-8), mutation counts, CALML6: putative copy-number alterations from GISTIC, 1p: putative copy-number alterations from GISTIC. The copy number is positively correlated with mRNA expression, while Admixture% EUR: Genetic Ancestry is positively correlated, and negatively correlated with phosphoprotein site level expression data by CPTAC. Then, we presented the statistical results of the proportions of different types of genomic alterations in each gene in the pan-cancer samples (Fig. 11B). The copy number of CALML6 gene was significantly correlated with mRNA, mutation counts, putative copy-number alterations from GISTIC, 1p: putative copy-number alterations from GISTIC, Admixture% EUR: Genetic Ancestry, phosphoprotein site level expression data by CPTAC, and methylation (Fig. 11C-I). Together, these findings provide a genomic overview of CALML6 alterations and their associations across multiple cancer types.

### qRT-PCR

**Fig. 12:**
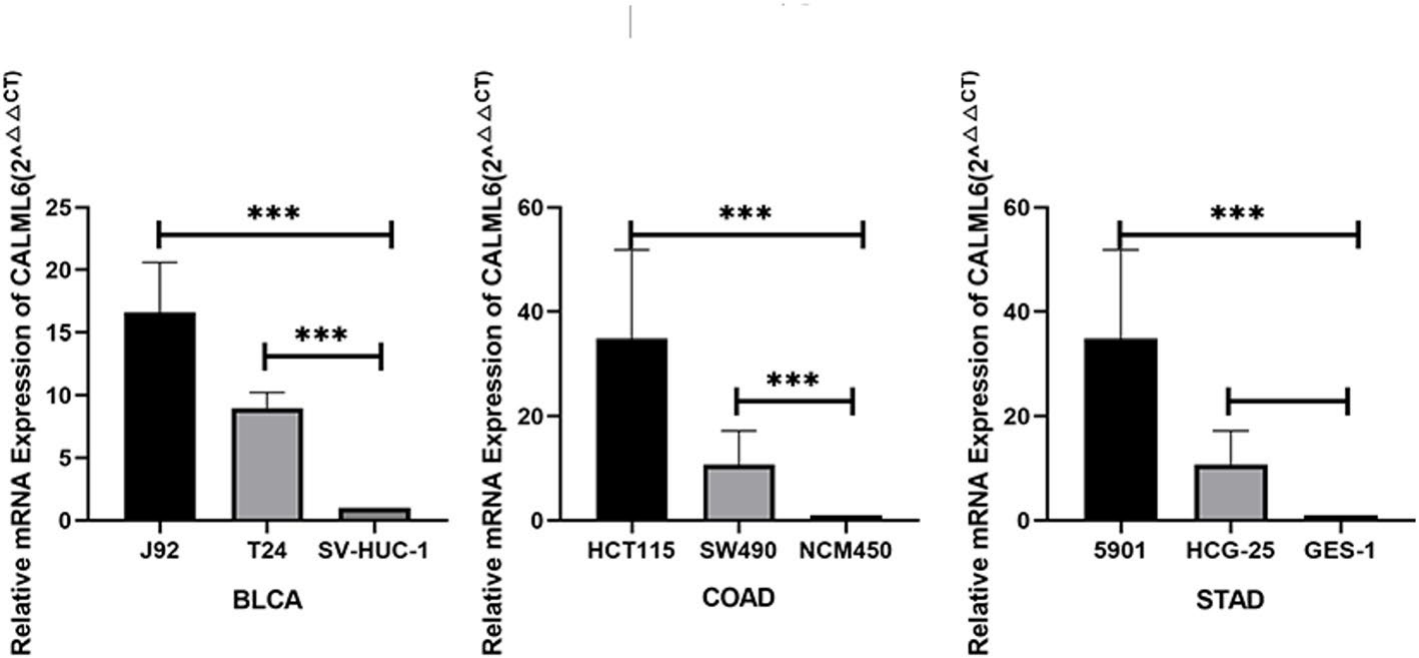
Relative mRNA expression of CALML6 in BLCA, COAD, STAD

CALML6 expression in BLCA, COAD, and STAD cell lines was evaluated by qRT-PCR. Based on the 2^-ΔΔCt analysis, CALML6 expression was higher in these cancer cell lines than in the corresponding normal cell lines (Fig. 12).

### Dependency landscape and mechanistic validation of CALML6

**Fig. 13:**
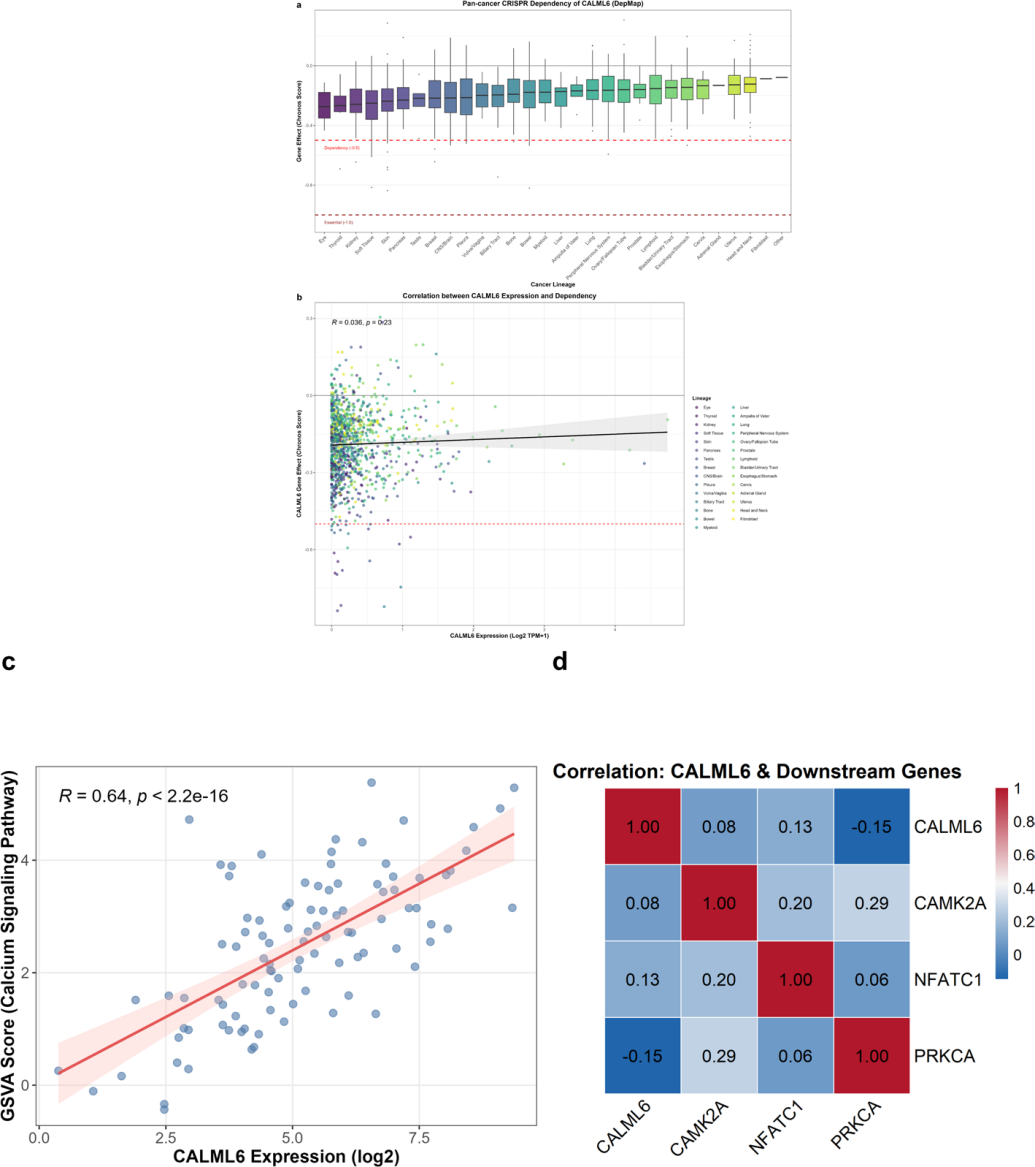
Functional characterization and mechanistic analysis of CALML6. **a** Distribution of CALML6 CRISPR gene effect scores (Chronos) across cancer lineages from DepMap. The red dashed line (-0.5) marks the dependency threshold. **b** Scatter plot showing the correlation between CALML6 expression and its gene dependency score. **c** Correlation analysis between CALML6 expression and the GSVA-derived Calcium Signaling Pathway activity score. The strong positive correlation (Spearman *R* = 0.64, *p* < 2.2e-16) indicates that CALML6 levels reflect pathway activation status. **d** Heatmap displaying the Spearman correlation coefficients between CALML6 and key downstream genes (CAMK2A, NFATC1, PRKCA), illustrating the specific molecular co-expression landscape

Pan-cancer CRISPR screening analysis revealed that CALML6 exhibits distinct lineage-specific dependency, particularly in Eye, Thyroid, and Kidney cancers, although it is not a universally essential gene (Fig. 13a). Notably, CALML6 dependency scores were not significantly correlated with its mRNA expression levels (Fig. 13b), suggesting that expression abundance alone does not dictate dependency. To explore the underlying mechanism, we integrated pathway activity scoring. GSVA results demonstrated a robust positive correlation between CALML6 expression and overall calcium signaling pathway activity (Fig. 13c), suggesting that CALML6 expression is associated with pathway activation status. Interestingly, subsequent analysis of individual downstream effectors (CAMK2A, NFATC1, PRKCA) revealed weaker linear correlations (Fig. 13d). This discrepancy suggests that CALML6 may be associated with calcium signaling activity at the network level, whereas correlations with individual downstream targets appear weaker and may involve more complex, non-linear relationships.

## Discussion

Recent pan-cancer studies have increasingly focused on identifying cancer-related genes with potential prognostic and functional relevance, including glycosylation-related genes in pancreatic ductal adenocarcinoma [11], while calcium-binding proteins have also been implicated in cancer progression, metastasis, and therapeutic targeting [12]. Cancer patients may also present with complex comorbid conditions, such as chronic kidney disease, which should be considered when interpreting cancer-related molecular findings and therapeutic implications [13]. Methodologically, reproducible large-scale biomedical analysis frameworks [14] and computational approaches for estimating tumour purity, stromal components, and immune cell admixture from expression data [15] provide important support for integrative pan-cancer analyses. Calcium signaling is a fundamental regulator of tumor growth, survival, and stress adaptation, providing a biologically sound framework for interpreting the pan-cancer relevance of CALML6 [16, 17]. The immune infiltration results support this view, because integrative bioinformatic approaches can capture stable differences in local immune composition and help explain context dependent gene effects across diseases [18].

This pattern may be of potential clinical interest, as current models for predicting response to immune checkpoint blockade increasingly incorporate combined molecular and immune features rather than any single variable alone [19]. The positive association of CALML6 with Tem and NK CD56bright cells, together with its negative association with iDC, Th1 cells, and neutrophils, suggests a broader shift in immune architecture within the tumor microenvironment [20]. That interpretation is consistent with recent clinical evidence showing that the baseline immune state of the tumor strongly influences the efficacy of immune checkpoint therapy [21]. The observed correlation between CALML6 expression and drug sensitivity suggests a possible association with treatment-response-related phenotypes, although this requires validation in treatment-annotated cohorts, which is in line with the current development of targeted agents and antibody based therapies in oncology [22].

This effect may be particularly meaningful in LGG, where immune cell related signatures have already shown value for prognostic stratification and may provide a more informative framework for evaluating CALML6 [23]. Our genomic analyses extend this interpretation, since the links among copy number variation, methylation, mutation related features, and mRNA expression support a multi level regulatory mechanism for CALML6 across cancers [24]. The inverse association with methylation is also notable, because epigenetic regulation is a recognized determinant of tumor phenotype and may contribute to the divergent clinical roles of CALML6 in different tumor types [25]. The CRISPR data provide dependency-related evidence, as the lineage-specific dependency pattern suggests that CALML6 is not universally essential but may represent a selective vulnerability in specific cancer settings [26]. The strong association between CALML6 and global calcium signaling activity, together with weaker correlations with individual downstream genes, favors a network level interpretation rather than a simple linear pathway model [27].

The immune significance of our findings is further supported by recent evidence that activated CD4 positive memory T cells and CXCL13 can enhance antitumor immunity, which strengthens the relevance of the CALML6 related Tem signal observed in this study [28]. Methodologically, the present work gains strength from integrating transcriptomic profiling, survival analysis, immune deconvolution, genomic characterization, drug sensitivity assessment, qRT-PCR validation, and dependency evidence within one pan cancer framework, although its retrospective design and limited experimental validation still require further confirmation in prospective and mechanistic studies [29]. The limitations of this study should be acknowledged. First, the analyses were based primarily on public databases, and differences in preprocessing procedures, sample composition, and data quality across platforms may have introduced bias. Second, no independent external pan-cancer cohort was included in the present study; therefore, the reproducibility of the observed trends still requires further validation. The current conclusions are mainly supported by convergent observations across multiple public databases and complementary analytical modules. Third, although qRT-PCR validation and dependency-related analyses were included, direct mechanistic experimental validation is still lacking. Accordingly, the biological and clinical implications of CALML6 should be interpreted with appropriate caution. Although CALML6 was associated with survival outcomes, immune infiltration, and drug sensitivity in the present study, its incremental value relative to established biomarkers and routine clinicopathological parameters was not evaluated. In addition, therapeutic response data were not incorporated. Therefore, the clinical applicability of CALML6 should be interpreted cautiously and requires further validation in clinically annotated and treatment-stratified cohorts [30–32].

## Conclusion

This study provides a comprehensive analysis of CALML6 across multiple cancer types and supports its potential relevance as a prognostic biomarker candidate. By integrating CRISPR-Cas9 screening and pathway profiling, we found that CALML6 exhibits lineage-specific dependencies and is associated with calcium signaling pathway activity. These findings provide a basis for future studies to further validate the biological relevance of CALML6 experimentally and to assess its potential clinical significance in cancer.

## Data Availability

The datasets analyzed in this study are publicly available in online repositories. TCGA (The Cancer Genome Atlas): RNA-seq gene expression data (processed via the STAR workflow) were downloaded from the GDC Data Portal ([https://portal.gdc.cancer.gov/](https:/portal.gdc.cancer.gov)). Transcripts Per Million (TPM) values from 33 cancer types were analyzed, including TCGA-ACC, TCGA-BLCA, TCGA-BRCA, TCGA-CESC, TCGA-CHOL, TCGA-COAD, TCGA-DLBC, TCGA-ESCA, TCGA-GBM, TCGA-HNSC, TCGA-KICH, TCGA-KIRC, TCGA-KIRP, TCGA-LAML, TCGA-LGG, TCGA-LIHC, TCGA-LUAD, TCGA-LUSC, TCGA-MESO, TCGA-OV, TCGA-PAAD, TCGA-PCPG, TCGA-PRAD, TCGA-READ, TCGA-SARC, TCGA-SKCM, TCGA-STAD, TCGA-TGCT, TCGA-THCA, TCGA-THYM, TCGA-UCEC, TCGA-UCS, and TCGA-UVM.Genomic data were accessed via the cBioPortal for Cancer Genomics ([https://www.cbioportal.org/](https:/www.cbioportal.org)), using the pancan\_atlas\_2018 dataset.Protein–protein interaction networks were analyzed using the STRING database ([https://string-db.org/](https:/string-db.org) , version 12.0).Outcome and drug sensitivity analyses were performed using the GSCA platform ([http://bioinfo.life.hust.edu.cn/GSCA/#/](http:/bioinfo.life.hust.edu.cn/GSCA)). Additional validation data were obtained from the UCSC Xena platform ([https://xena.ucsc.edu/](https:/xena.ucsc.edu)), TCGA Pan-Cancer (PANCAN) dataset.All experimental materials used for qRT-PCR validation were obtained from commercial suppliers.
